# Case report: A case of intercostal intramuscular hemangioma with sternal invasion

**DOI:** 10.3389/fonc.2024.1360626

**Published:** 2024-10-01

**Authors:** Dongdong Liu, Yuchen Bao, Yanzhe Wang, Xiangjun Lu, Jian Shen, Siyuan Huang, Ming Zhao, Haitao Wang

**Affiliations:** ^1^ Cancer Center, Department of Thoracic Surgery, Zhejiang Provincial People’s Hospital, People’s Hospital of Hangzhou Medical College, Hangzhou, Zhejiang, China; ^2^ Hangzhou Normal University, Hangzhou, Zhejiang, China; ^3^ Zhejiang Chinese Medical University, Hangzhou, Zhejiang, China; ^4^ Department of Radiology, Zhejiang Provincial People’s Hospital, People’s Hospital of Hangzhou Medical College, Hangzhou, China; ^5^ Department of Pathology, Zhejiang Provincial People’s Hospital, People’s Hospital of Hangzhou Medical College, Hangzhou, China

**Keywords:** intercostal, intramuscular hemangioma, sternal, invasion, case

## Abstract

**Background:**

Intramuscular hemangioma is a vascular malformation occurring in muscle tissues. It is most common in skeletal muscles of limbs, especially lower limbs in childhood. The intercostal intramuscular hemangioma with sternal invasion is very rare.

**Case presentation:**

The 47-year-old female patient was hospitalized due to “suddenly chest pain for 4 days”. Chest contrast-enhanced CT indicated bony changes in the sternum, accompanied by soft tissue shadows in the posterior sternum and diaphragmatic angle. After admission, physical examination showed: peristernal tenderness, percussion pain, other no obvious positive signs. PET-CT examination indicated that there was a high possibility of benign sternal changes. No cancer cells were found by sternal puncture. Surgical biopsy was performed and pathological findings were consistent with intramuscular hemangioma.

**Conclusion:**

Intramuscular hemangioma with bone invasion is very rare, imaging examination is difficult to indicate obvious evidence, preoperative diagnosis is difficult, complete surgical resection is very important, more personalized treatment should be provided according to the overall situation of the patient.

## Background

Hemangioma is a common benign tumor, mostly in childhood (1). Intramuscular hemangioma (IMH) refers to a class of mass that is primary in skeletal muscle and characterized by abnormal proliferation of blood vessels in muscle tissue, which accounts for less than 1% of all hemangiomas (2, 3). IMH is a rare disease, most common in the skeletal muscles of the limbs, the lower extremities and the thighs (36%), followed by the calves (17%) (4). Because it has no obvious special clinical symptoms, low incidence and little clinical cognition, it is easy to be missed or misdiagnosed. However, the occurrence of intrastinal intercostal hemangioma with sternal invasion is more rare, and there were few similar cases reported before this report.

## Case presentation

**Figure 1:**
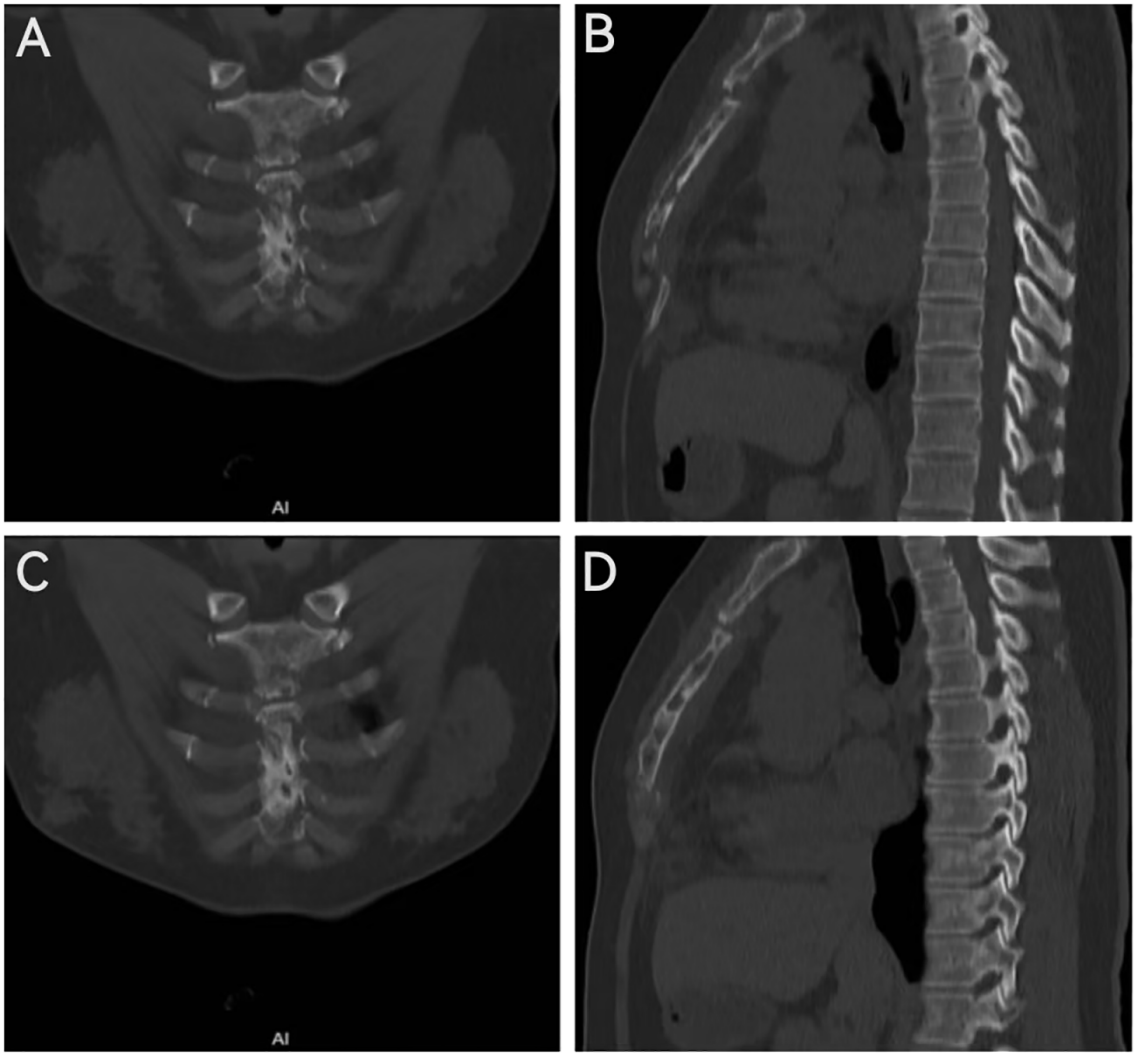
Chest enhanced CT coronal view **(A, C)**; sagittal view **(B, D)**.

The 47-year-old female patient was admitted to hospital with “suddenly chest pain for 4 days”. Enhanced CT scan of the chest reveals alterations in the sternum, characterized by soft tissue opacities at the posterior margin of the sternum and cardiac diaphragmatic angle. The enhanced images demonstrate mild to moderate enhancement within these regions. There is an uneven reduction in sternal bone density, accompanied by subluxation at the sternal angle (**Figure 1**).

**Figure 2:**
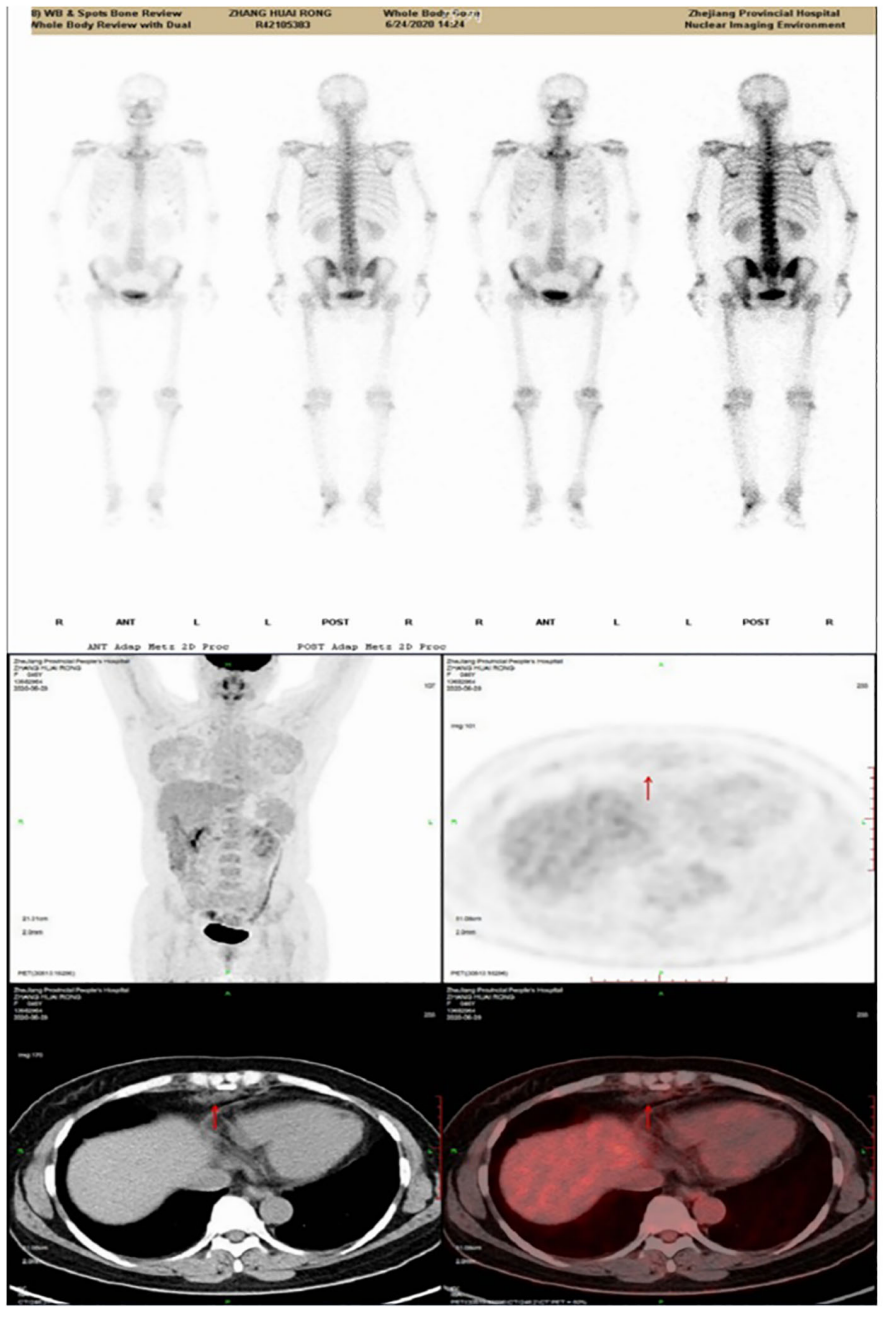
PET/CT scan image.

The patient has no history of trauma, respiratory diseases or other special diseases. After admission, physical examination showed: peristernal tenderness, percussion pain, other no obvious positive signs. Serum carbohydrate antigen-125:37.2U/mL, other test indicators showed no obvious abnormalities. Considering the possibility of malignant tumor. The PET-CT examination identified sternal injury presenting as visible lytic bone lesions. Soft tissue density shadows were detected at the posterior margin of the sternum, bilateral intercostal spaces, and right diaphragmatic angle, exhibiting respective SUVmax values around 2.52 and 2.2. The diagnosis indicates ossifying sternal injury accompanied by mildly elevated FDG metabolism within these soft tissue density shadows in the mentioned areas; these observations potentially suggest benign lesions (**Figure 2**).

**Figure 3:**
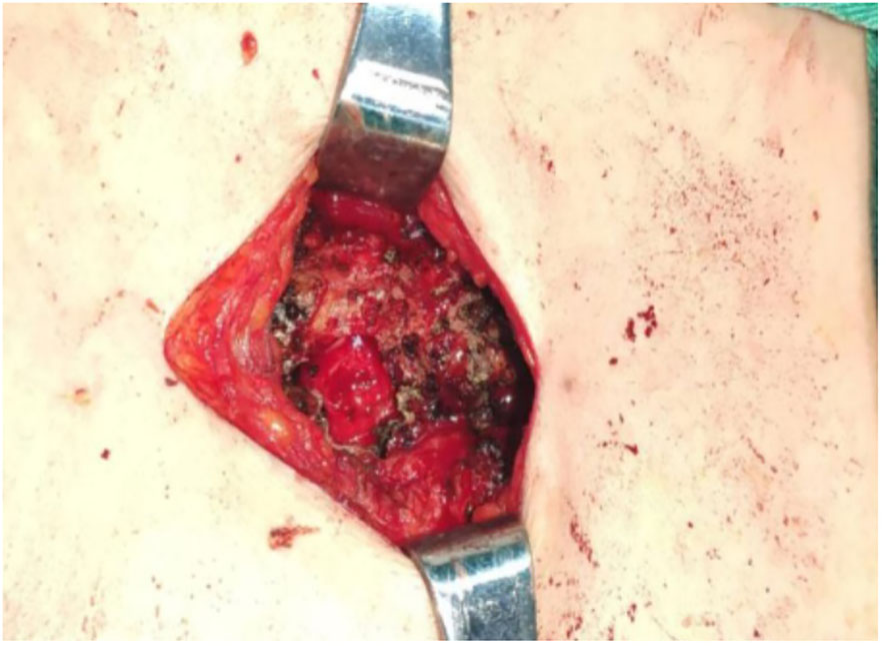
Intraoperative images.

The results of sternal puncture biopsy indicated that no cancer cells were found on “sternal tumor puncture fluid based smear”. Combined with the above examinations, complete surgical resection of the patient was more invasive. In order to confirm the diagnosis, the sternum and soft tissue biopsy under general anesthesia was performed on July 9, 2020. Intraoperative: no obvious mass like tissue was found in front of the sternum, but the sternal cortex showed the changes like worm eating, bone marrow cavity showed cavity changes, and chyloid-like fluid outflow was observed (**Figure 3**).

**Figure 4:**
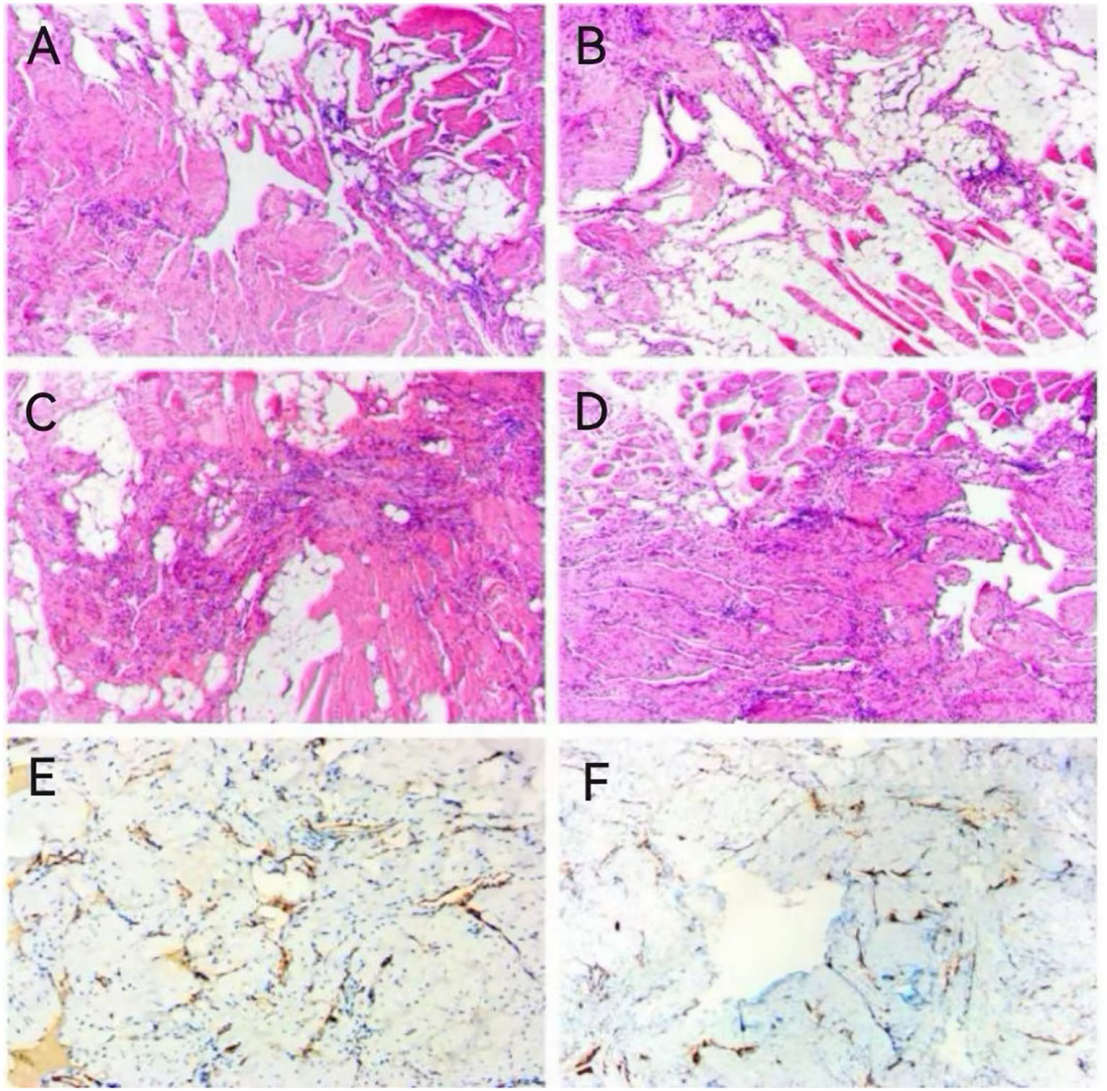
HE staining results **(A–D)**; Immunohistochemical staining results **(E, F)**.

Intraoperative freezing was observed: disorganized proliferation of spindle cells in the striated muscle, fibrotic or neurofibrotic neoplastic lesions were considered. Postoperative routine pathology: 1.the lesion was poorly circumscribed and composed of collections of abnormal veins of varying size lined by bland, markedly attenuated endothelium. These clustered vessels were interspersed in the muscles and adipose tissues. Immunohistochemical staining of CD31 decorated the attenuated endothelium 2. Immunohistochemical staining results: CD31, CD34 (+) (+), SMA (+), Desmin (–), according to (–), SOX10 (–) and Ki67 (about 1%) (**Figure 4**).

## Discussion and conclusions

The term intramuscular hemangioma was proposed by the World Health Organization and named by the International Association of Vascular Anomalies in 2018. It is mostly a vascular malformation occurring in muscle tissue, rather than a vascular tumor in the true sense (5). Most intramuscular hemangiomas occur in childhood, but because most intramuscular hemangiomas occur in deep tissues, they are not easy to be found in childhood, and are often diagnosed in early adulthood. It has been reported that more than 94% of cases are found before the age of 30 (1, 6, 7). Previous reports indicate that intramuscular hemangiomas are more common in women (8), but there have been reports to the contrary (9). Although this case is also a female patient, the center believes that the pathogenesis is not clear, combined with the characteristics of hemangioma, it does not indicate that the incidence of this disease is gender-related.

The clinical manifestations of intramural hemangioma are not typical. Common hemangioma in skeletal muscle of lower limbs can be manifested as local swelling and pain, which gets worse after exercise and is mostly caused by pain caused by muscle tissue compression (10). In this case, the patient was accompanied by sternal invasion. However, combined with the medical history, intraoperative manifestations and postoperative pathological reports, it was considered that the pain might be caused by peripheral tissue or intercostal nerve compression caused by mass enlargement, or bone pain caused by sternal invasion.

The Ultrasonography (US) and magnetic resonance imaging (MRI) are considered to provide evidence for the diagnosis of an intramuscular hemangioma, and MRI provides a clear indication of the lesion’s size, adjacence to adjacent tissues, tumor interior, and vasculature (11–13). Some articles believe that most intramuscular hemangiomas can be diagnosed only by imaging and clinical manifestations, without the need for puncture or soft tissue biopsy (14). However, it has also been reported that only 8% of cases of intramuscular hemangioma can be clearly diagnosed before surgery due to the atypical clinical symptoms and low cognition of the disease (15). As some studies have pointed out, MRI alone is not appropriate when intramuscular hemangiomas are associated with bone invasion (16, 17). Combined with the characteristics of this case, the center believes that the preoperative imaging, especially the application of MRI, is of great help to the diagnosis of some typical intramuscular hemangiomas of skeletal muscle of lower limbs. But in a rare and unusual case like this one, imaging alone, or even biopsy alone, cannot confirm the diagnosis.

In this case, the CT findings on admission indicated bony changes in the sternum. It increased the suspicion of primary or metastatic malignant tumors, but after PET-CT examination, it was suggested that FDG metabolism was slightly increased in the lesion, which might be considered as benign lesions. Therefore, it is necessary for clinicians to have a wider understanding of all aspects of knowledge and to discuss multidisciplinary team (MDT) in the early stage.

Intramuscular hemangioma is a benign mass, but cases with surrounding bone tissue invasion have been reported, including local osteopecia, cortical thickening, trabecular coarsening, and surrounding bone erosion (18–20). However, the current mechanism of intramural hemangioma bone invasion is not yet clear. However, current studies indicate that the surface of hemangioma is accompanied by vascular endothelial growth Factor receptor-2 (VEGFR-2), and the abnormal expression of VEGFR-2 is related to the abnormal growth of part of the blood vessels inside the hemangioma (21, 22). Meanwhile, studies have pointed out that VEGF plays an important role in bone remodeling (23). Therefore, this factor may become the future research direction.

Various treatment modalities are available for intramuscular hemangioma, including steroid injections, radiotherapy, sclerosing agent injections, and surgical intervention; however, surgical treatment remains paramount. Nevertheless, due to the tumor’s size and its infiltration of adjacent blood vessels, nerves, and tissues around the sternum as well as the potential risks associated with radical surgery-induced unexpected injuries, achieving complete margin integrity for eradication poses significant challenges. In fact, the status of surgical margins is a crucial determinant influencing postoperative recurrence rates in intramuscular hemangioma cases. According to statistics (24, 25), within 5 years after local excision alone, recurrence rates can reach up to 30%. Therefore, the primary objective should focus on alleviating local symptoms caused by tumor compression through surgery while ensuring accurate diagnosis rather than considering complete tumor removal as the preferred treatment approach.

Through our treatment, we have successfully clarified the patient’s diagnosis and effectively ruled out the possibility of a malignant tumor, thereby alleviating the patient’s anxiety. Simultaneously, while aiming to delay further progression of the condition as much as possible, we exercised caution in not excessively resecting the lesion to ensure optimal postoperative quality of life. However, it is important to acknowledge certain limitations in this approach: namely, failure to consider the potential presence of intramuscular vascular malformation prior to surgery and lack of preoperative MDT discussion. In future similar cases, improvements should be made in MRI and US examinations along with timely initiation of preoperative MDT discussions when contemplating the likelihood of intramuscular vascular malformation.

Patient perspective: Our treatment intervention facilitated the patient’s diagnostic clarification and effectively ruled out the presence of malignant tumors, thereby alleviating their psychological distress and enhancing their comprehension of the condition. Simultaneously, considering the incurable nature of this disease, our patient-centered treatment plan prioritizing quality of life was perceived by the patient as the optimal choice available.

To sum up, intramuscular hemangioma with bone invasion is a rare disease with little cognition at present. Preoperative diagnosis is still the biggest challenge. Complete surgical resection is essential. However, multidisciplinary discussions should be conducted at an early stage according to the patient’s overall situation to provide a more personalized treatment plan.

## Data availability statement

The original contributions presented in the study are included in the article/supplementary material. Further inquiries can be directed to the corresponding author.

## Ethics statement

Written informed consent was obtained from the individual(s) for the publication of any potentially identifiable images or data included in this article.

## Author contributions

DL: Writing – original draft, Writing – review & editing, Investigation. YB: Writing – original draft, Writing – review & editing, Investigation. YW: Writing – review & editing, Investigation, Writing – original draft. XL: Writing – review & editing. JS: Writing – original draft. SH: Writing – original draft. MZ: Writing – review & editing. HW: Writing – original draft, Writing – review & editing.
